# An Automated, Clip-Type, Small Internet of Things Camera-Based Tomato Flower and Fruit Monitoring and Harvest Prediction System

**DOI:** 10.3390/s22072456

**Published:** 2022-03-23

**Authors:** Unseok Lee, Md Parvez Islam, Nobuo Kochi, Kenichi Tokuda, Yuka Nakano, Hiroki Naito, Yasushi Kawasaki, Tomohiko Ota, Tomomi Sugiyama, Dong-Hyuk Ahn

**Affiliations:** 1 Research Center for Agricultural Robotics, National Agriculture Food Research Organization (NARO), Tsukuba 305-0856, Japan; islam.md_parvez.by@ehime-u.ac.jp (M.P.I.); kochin817@affrc.go.jp (N.K.); k.tokuda@naro.affrc.go.jp (K.T.); yuka88@affrc.go.jp (Y.N.); naitoh758@naro.affrc.go.jp (H.N.); yask@naro.affrc.go.jp (Y.K.); toota@naro.affrc.go.jp (T.O.); 2 Institute of Vegetable and Floriculture Science, National Agriculture Food Research Organization (NARO), Tsukuba 305-8519, Japan; sugiyamat209@affrc.go.jp (T.S.); ahn@affrc.go.jp (D.-H.A.)

**Keywords:** horticulture, tomato cultivation, deep learning, harvest date estimation, flowers and fruits detection, internet of things, artificial intelligence camera

## Abstract

Automated crop monitoring using image analysis is commonly used in horticulture. Image-processing technologies have been used in several studies to monitor growth, determine harvest time, and estimate yield. However, accurate monitoring of flowers and fruits in addition to tracking their movements is difficult because of their location on an individual plant among a cluster of plants. In this study, an automated clip-type Internet of Things (IoT) camera-based growth monitoring and harvest date prediction system was proposed and designed for tomato cultivation. Multiple clip-type IoT cameras were installed on trusses inside a greenhouse, and the growth of tomato flowers and fruits was monitored using deep learning-based blooming flower and immature fruit detection. In addition, the harvest date was calculated using these data and temperatures inside the greenhouse. Our system was tested over three months. Harvest dates measured using our system were comparable with the data manually recorded. These results suggest that the system could accurately detect anthesis, number of immature fruits, and predict the harvest date within an error range of ±2.03 days in tomato plants. This system can be used to support crop growth management in greenhouses.

## 1. Introduction

Tomato flowers and fruits are important growth parameters for the calculation of the date of anthesis, the total number of fruits, and final yield [1]. The yield is an import factor for the production planning and management of greenhouses, which can be calculated by the temperature if the date of anthesis can be screened and tracked to determine fruiting on each tomato truss.

However, manual screening of tomato flowers and fruits on the truss in a cluster of plants is time-consuming because of plant density, the complexity of structure (such as overlapping, twisted, irregular movements of position), and unconstrained environment (such as colors, shapes, and sizes) [2]. In addition, identification and tracking of flowers and fruits become difficult because the complexity of tomato structure increases significantly as the tomato plant grows.

Recently, several studies have reported flower and fruit detection using automated systems. Automated counting techniques have been developed for several plants, such as apples [3,4,5], citrus [6,7], dragon fruits [8], mangoes [9], peppers [10], and strawberries [11]. Some of these studies employed image processing technologies to detect and count flowers and fruits using color spaces such as hue–saturation–value (HSV) [4], RGB, and YCbCr [8]. Zhao et al. [12] detected mature tomato fruits using color analysis with Haar-like features and an AdaBoost classifier, while Liu et al. [13] applied color analysis with the histogram of oriented gradients (HOG) and support vector machine (SVM). Deep learning has also been employed in numerous studies for detecting fruits and flowers of plants, as demonstrated by Sa et al. [14], Rahnemoonfar and Sheppard [15], Dias et al. [16,17], Sun et al. [18], and Chen et al. [11]. A frequently used algorithm applied to detect fruits in natural environments is a faster region convolutional neural network, or Faster R-CNN [11,14,18,19], while another study [17] introduced the detection of flowers using convolutional neural networks (CNN) with SVM. However, as these methods are not detection algorithms or image acquisition methods that can be responded or modified as the complexity of crops increases, they have limitations in tracking specific tomato flowers and fruits of a single tomato plant when the complexity of the tomato structure increases significantly over time in fields or greenhouses.

Eizentals [20] developed an automatic picking robot for a pepper greenhouse that has sensors to recognize peppers, algorithms to grab peppers, and a movement system. Yuan et al. [21] developed a pollination robot for hormone treatment by detecting tomato flower trusses using 3D information based on stereoscopy. Additionally, Seo et al. [22] proposed a monitoring robot system for tomato fruits using deep learning-based detection. Robot-based flower and fruit detection and tracking can be a flexible system to respond to the increasing complexity of tomato structure. A robot can help in the visualization of tomato flowers and fruits to detect and track those that cannot be clearly visualized due to leaves and branches (e.g., lifting leaves). However, it is significantly difficult to not cause damage to the stem, leaf, or fruit of tomatoes using such robots. Additionally, a high-cost is required to install such robots on plants.

Here, we propose an effective clip-type IoT camera-based tomato growth monitoring and harvest prediction system, which consists of a clip and an IoT camera-based tomato flower and fruit detection and tracking system to predict time of harvest. Clips are frequently used in greenhouse horticulture as they are used to fix the tomato stem with a guide string. The clip-type IoT camera is installed with a guide string to the tomato stem and it captures the images of fruits and flowers of the truss at close intervals. The camera was designed to be easily installed on a truss, which solves the identification and tracking problems mentioned above as it moves along with the growing tomato stem. The clip-type IoT camera captured each truss image and tracked blooming flowers to immature fruits in the truss using deep learning-based object detection and tracking algorithms. In addition, the harvest date could be calculated using the date of anthesis (i.e., blooming flower detection and tracking from blooming flower to immature fruit) and integrated daily average temperature forecasting inside the greenhouse, since the temperature influences growth and development of the tomato plant [23,24]. To evaluate the performance of our system, the clip-type IoT cameras were installed on four tomato plants in our greenhouse (Tsukuba, Ibaraki, Japan). Our system detected and tracked the flowers and fruits of each tomato truss for three months. Three clip-type IoT cameras were used per tomato plant based on the growth rate of tomatoes. The top three trusses from the growing tip were monitored. Additionally, the harvest date predicated by our system was compared with the data measured manually. The results showed an error of ±2.03 days. The results of this study show that our system can automatically predict tomato harvest date with high performance using detection and tracking of flowers and fruits. The prediction can be used as an indicator for crop growth and harvest management in greenhouses.

## 2. Materials and Methods

### 2.1. System Overview

#### 2.1.1. System Pipeline

**Figure 1:**
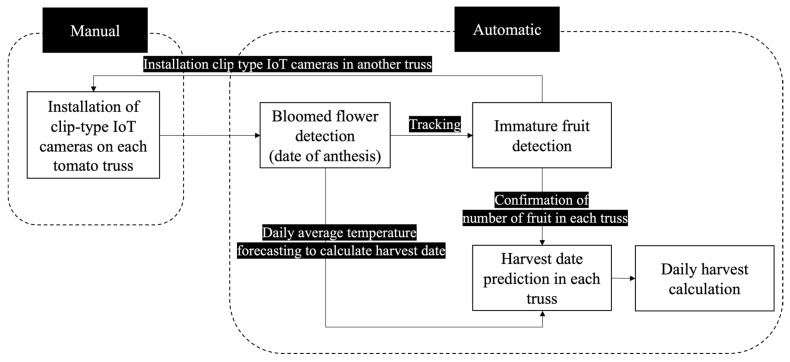
Pipeline of the system for tomato cultivation. IoT: Internet of Things.

Our system was designed to predict the harvest date by tracking the detected blooming flowers to immature fruit and temperature forecasting (Figure 1). First, we installed self-designed clip-type IoT cameras on each tomato truss manually. After installation, the system acquired images of each truss every minute, and blooming flowers were detected in each image. Then, the system calculated harvest date using daily average temperature forecasting from the anthesis date if bloomed flowers were detected; the anthesis date is the date when the system detects a blooming flower in the truss. The system tracked each detected blooming flower until the formation of an immature fruit to confirm the number of fruits that can actually be harvested for each truss; some blooming flowers do not form fruit; however, most immature fruit can be harvested. Finally, the system calculated daily harvest using the harvest date prediction and confirmation in each truss.

#### 2.1.2. System Configuration

**Figure 2:**
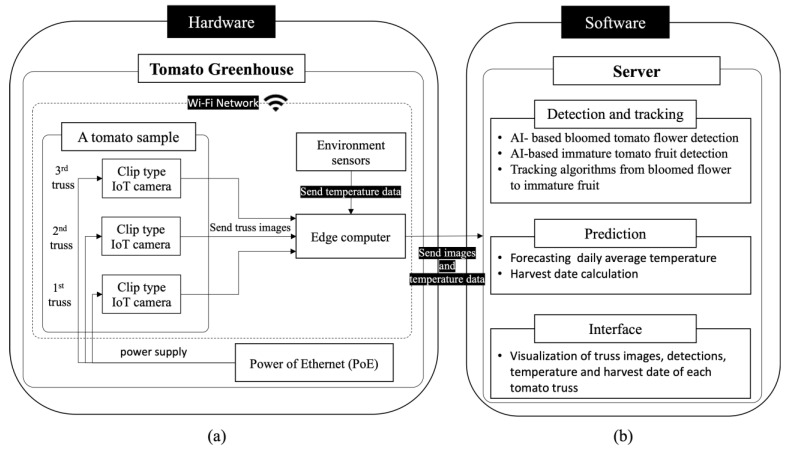
Configuration of system. (**a**) hardware configuration; (**b**) software configuration.

Our system consisted of clip-type IoT cameras, an edge computer, a Power over Ethernet (PoE), and environment sensors in the hardware (Figure 2a). Three clip-type IoT cameras were used per tomato plant. The cameras use electrical power from the PoE and connect wirelessly to the edge computer of the greenhouse (Raspberry Pi) via an internal network (i.e., Wi-Fi). Environmental sensors were connected to the edge computer as well. Each clip-type IoT camera sent the captured images of the tomato truss to the edge computer, and the sensor sent temperature data. All data were stored in the edge computer.

The system also consists of pre-trained artificial intelligence-based tomato flower and immature fruit detection methods, tracking algorithms from bloomed flowers to immature fruit, daily average temperature prediction, and rule-based harvest date calculation. In addition, the system includes an interface that can visualize each result (Figure 2b).

#### 2.1.3. Clip-Type IoT Camera Design

**Figure 3:**
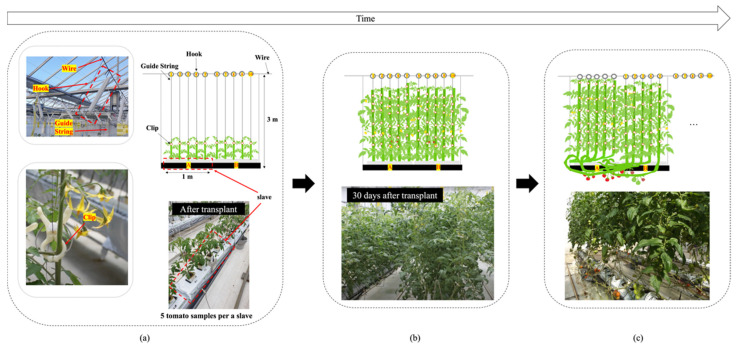
Complexity of tomato structure over time. (**a**) low complexity of tomato structure; (**b**) high complexity of tomato structure to track specific flowers, fruit, and ripe tomato samples because the number of invisible tomato flowers and fruit increased; (**c**) highest complexity of tomato structure because the hooks are moved in irregular positions and directions.

**Figure 4:**
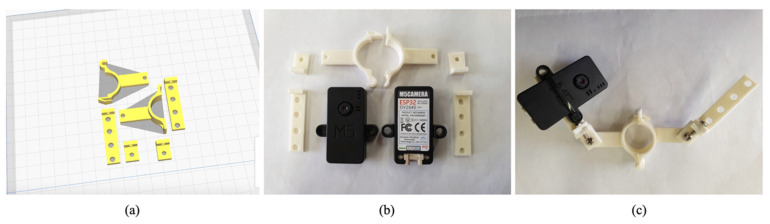
Self-designed clip-type IoT camera. IoT: Internet of Things. (**a**) A 3D model of the clip; (**b**) A printed clip and IoT cameras; (**c**) A clip-type IoT camera.

**Figure 5:**
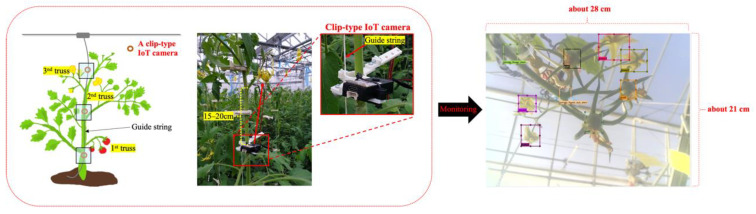
Installation of clip-type IoT cameras. IoT: Internet of Things.

The complexity of tomato fruit structure increased significantly over time in the greenhouse (Figure 3). At the beginning (e.g., between transplant and about 30 days after transplant), it is simple to track flowers and fruits; however, when they become invisible or move irregularly (Figure 3b,c), it becomes very difficult to track the specific flower, fruit, and tomato samples. The clip-type IoT camera design was inspired by the movement of clips with the tomato stem, even when the position of the tomato stem or truss was changed because of its growth and development, and other agricultural works in horticulture greenhouses. In other words, the clip-type camera can continuously monitor specific flowers and fruits when it is fixed near them (i.e., to the truss). The customized clip was created based on the existing 3D model of the clip (https://www.instructables.com/3D-Printed-Trellis-Clip-designed-in-Fusion-360, accessed on 10 March 2022). Two arms were added to the 3D model of the clip to install the IoT camera on the clip, as shown in Figure 4a. The two arms on each side of the clip are designed not only to maintain the balance of the clip, but also to mount additional sensors or batteries. In addition, each arm consists of two joints to adjust the position of the IoT camera lens according to the initial position of each flower and fruit, and each joint was fixed with screws. The clip-type IoT camera was installed with a tomato stem and a guide string on the bottom of each truss (Figure 5), with the camera lens facing upwards. M5Camera was used for the clip-type IoT camera (M5Stack, Shenzhen, China) as it is a modular and stackable toolkit based on ESP32 [25]. The camera is light and small with a durable case, OV 2640 lens (65° Field of View, maximum 1600 × 1200 resolution), USB Type-C power connector, and a Wi-Fi module. Thus, the camera was adopted for use with the customized clip because, even if it is fixed with the tomato stem, it puts less stress on the tomato plant. Moreover, it was designed to enable simple installation and removal by easily available tools in a greenhouse.

#### 2.1.4. Data Acquisition

**Figure 6:**
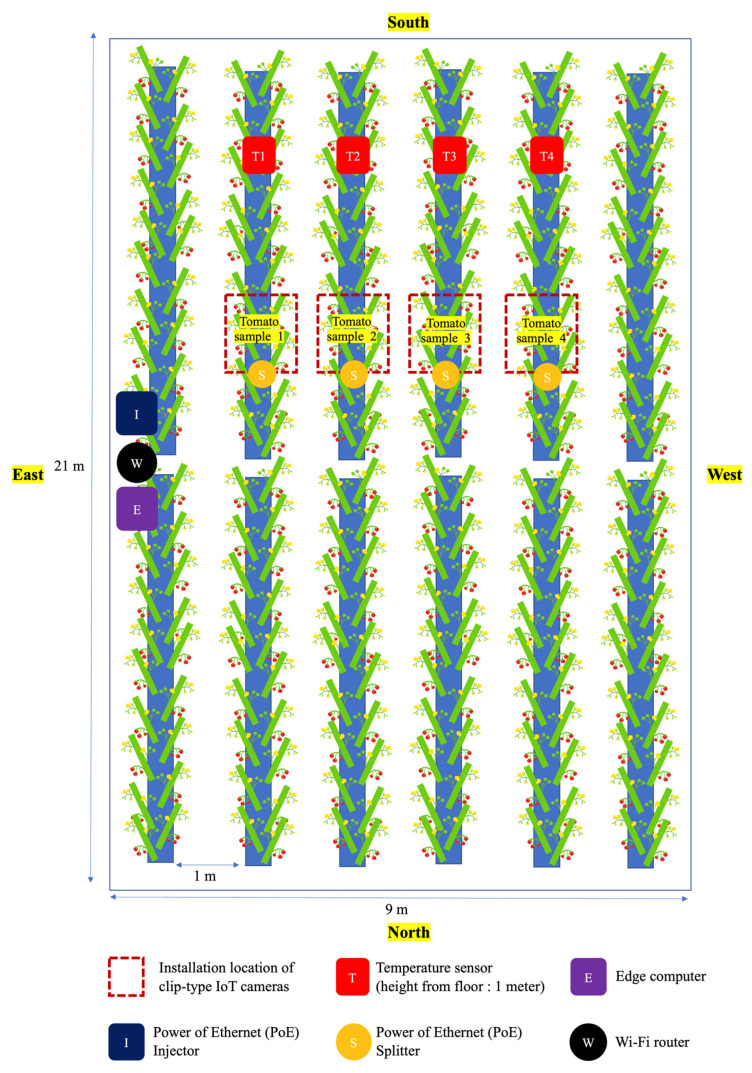
Layout of the experimental greenhouse.

A tomato greenhouse was prepared by the Institute of Fruit Tree and Tea Science, National Agriculture and Food Research Organization (NARO) in Tsukuba, Ibaraki, Japan (Figure 6). The tomato (*Solanum lycopersicum*) cultivar used in this study was the Japanese cultivar ‘Momotaro York’. Initial data of flowers and fruits were collected from November to mid-December 2019 as a preliminary experiment. Our system used the collected data to train a deep learning-based tomato flower and fruit detection model. We determined the most appropriate camera position and the number of clip-type IoT cameras required per tomato plant depending on the plant growth rate through this experiment. Tomato flowers and fruits of each truss were monitored. First, a camera was installed on the first truss after transplantation. The remaining cameras (i.e., two cameras) were installed when the second and third trusses were generated. Then, from the fourth truss, the clip-type IoT camera at the bottom was reused. Each clip-type IoT camera was installed on a tomato stem and a guide string together, and the cameras were placed under the truss. The distance between the camera lens and the target truss was set at 15 to 20 cm (Figure 5). This distance was based on our experiments, from which a single camera could monitor the growth of the flowers and fruits of the ‘Momotaro York’ cultivar. Image data of each truss were acquired from sunrise to sunset and measured every minute. The image data were acquired for each truss from the flower bud stage until all fruits were more than approximately 3 cm in diameter.

### 2.2. Deep Learning-Based Flower and Fruit Monitoring

#### Detection

**Figure 7:**
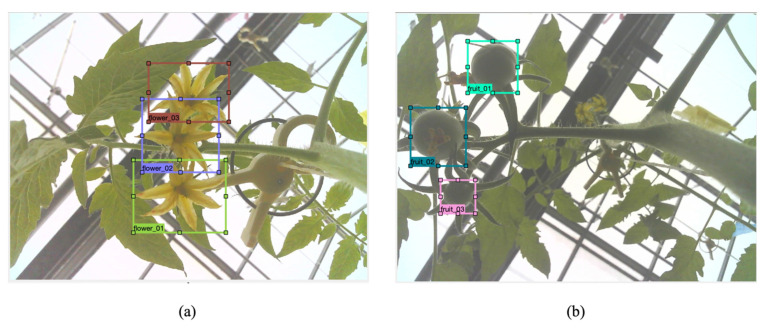
Deep learning-based bloomed flowers and immature fruits detection in tomato. (**a**) Bloomed flower detection; (**b**) Immature fruit detection.

Our system monitors flowers and fruits using deep learning-based flowers and fruits detection and tracking. The system detects tomato flowers and fruits of each truss. We defined two targeted object classes, including fully bloomed flowers and immature fruits, and we ignored the early bud regions and half-bloomed flowers in the flower images (Figure 7).

**Figure 8:**
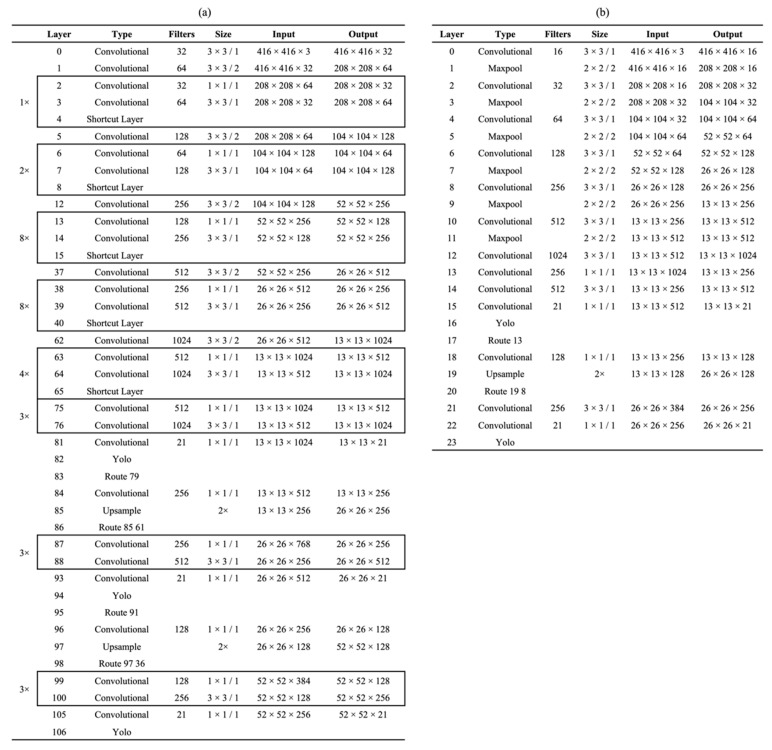
Deep-learning network architecture diagrams. (**a**) YOLOv3 network structure based on Darknet53; (**b**) YOLOv3-Tiny network structure.

The detection algorithm used in this study was based on the You Only Look Once (YOLO) model, including the YOLO v2 [26] and YOLO v3 [27] algorithms. The architecture of the YOLO deep learning networks is shown in Figure 8, where YOLO v3 included 106 layers that were larger than YOLO v3-Tiny, which had 23 layers. Object detection with this algorithm was accomplished with both speed and precision, where a single neural network predicts bounding boxes and class possibilities straight from the full image in a single evaluation. To detect the flower and fruit regions in the image, the coordinates of the flower and fruit targets in each image were trained and detected using the YOLO algorithm. We trained and compared the YOLO v2 and v3 models to determine the performance of each network and design for model compatibility with the clip-type IoT camera.

In this study, we utilized four evaluation indices to evaluate the network performance of flower and fruit detection, including precision, recall, F1 score, and mean Average Precision (mAP). This is considered the concept of Intersection over Union (IoU), which computes the intersection over the ground truth and the predicted bounding box. We considered an IoU threshold of 0.5 or 50%. If the IoU is greater than or equal to 0.5, object detection is classified as true positive (*TP*); otherwise, it is classified as false positive (*FP*). If the ground truth fails to be detected, it is classified as a false negative (*FN*). Precision, recall, and F1 score can be calculated as shown in Equations (1)–(3), respectively. The average precision (*AP*) shows the average accuracy in each class, as shown in Equation (4), finding the area under the precision–recall curve, while mAP was calculated for the entire dataset representing the accuracy of the detection model, where a higher value indicates a better detection. Equation (5) represents the mAP calculation, where *N* is the number of classes:

(1) $$Precision=\frac{TP}{TP+FP}$$

(2) $$Recall=\frac{TP}{TP+FN}$$

(3) $$F1\;score=2\cdot \frac{Precision\cdot Recall}{Precision+Recall}$$

(4) $$AP=\int_{0}^{1}p\left(r\right)dr,\;p\left(r\right):\mathrm{precision},\;r:\mathrm{recall}$$

(5) $$mAP=\frac{1}{N}\sum_{i=1}^{N}AP_{i}$$

### 2.3. Tracking

**Figure 9:**
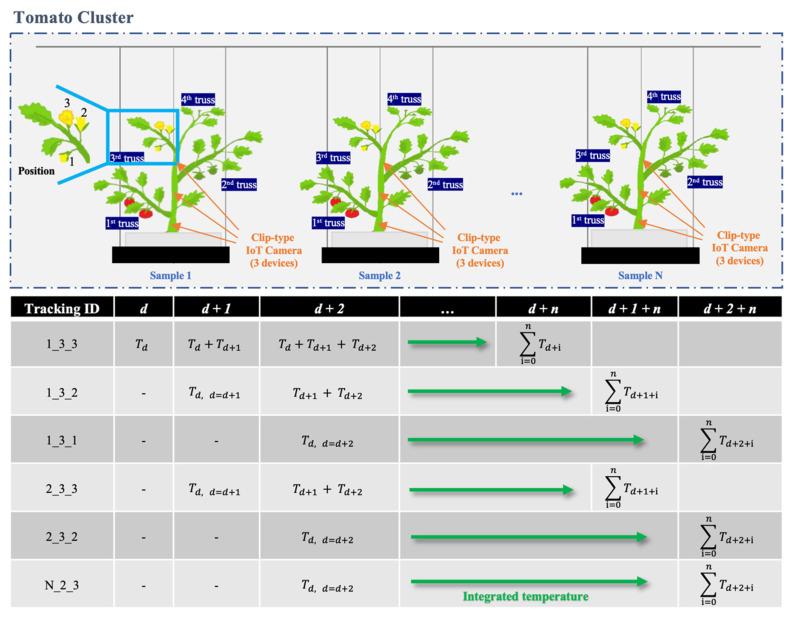
Tracking and harvest date estimation from integrated average daily temperature. Tracking ID format = Sample number_Truss number_Position number; *T* = Average of daily temperature (° Celsius); *d* = Date of flower opening; *d* + *n* = harvesting date at specific integrated average of daily temperature (e.g., $\overset{n}{\underset{i=0}{\sum }}T_{d+i}=1100{\;}^{{}^{\circ}}C$).

Our system tracked detected flowers and immature fruits using the coordinates of the detected boxes. First, the detected blooming flowers and fruits of each truss are defined with unique IDs, as shown in the ‘Tracking ID’ column of Figure 9.

**Figure 10:**
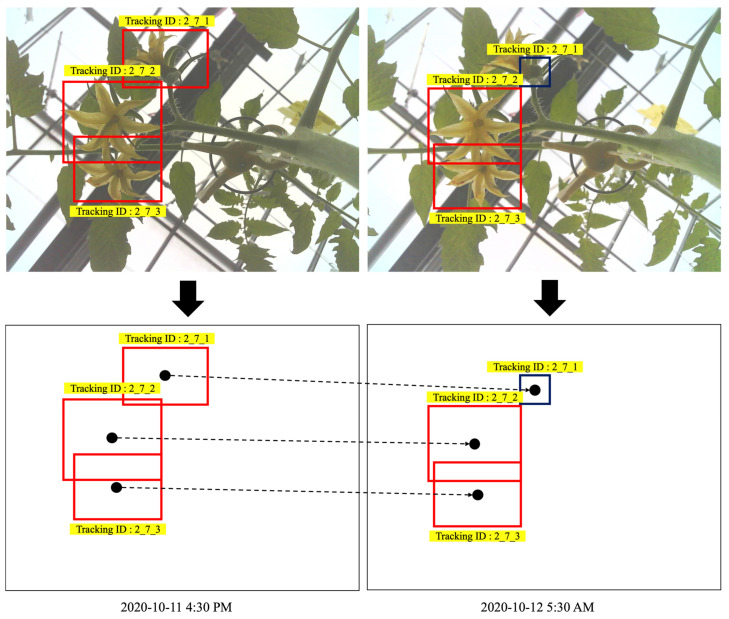
Bloomed flowers and immature fruits tracking in tomato.

**Figure 11:**
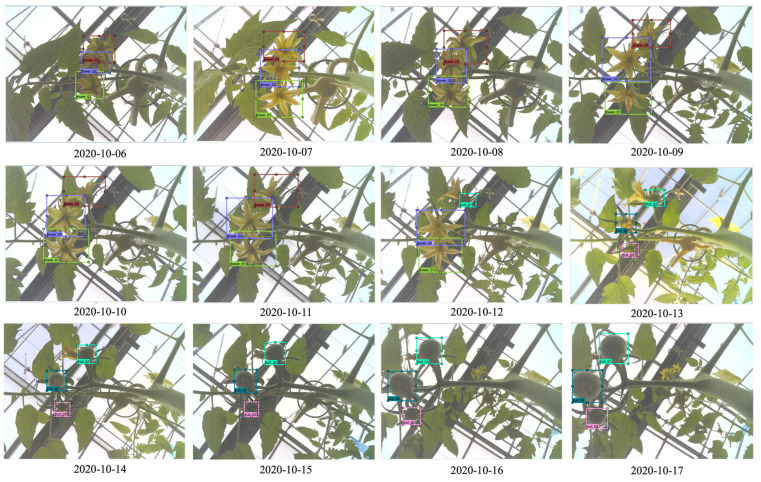
Time-series detection and tracking.

The tracking ID includes the monitored tomato plant ID, truss ID, and blooming flower ID in the truss being monitored. The detected flowers and fruits were tracked using the image data acquired every minute from sunrise to sunset; in some cases, the target (i.e., flowers and fruits on truss) or the camera is shifted due to agricultural work (e.g., pruning, harvest), thus the interval of data acquisition was set to be short for tracking. When data acquisition starts again at sunrise the next day, the positions of flowers and fruits may be shifted, or there may be newly blooming flowers or new fruits; often, the positions may be shifted by agricultural works in the greenhouse. Thus, our system detects blooming flowers and immature fruits in every captured image. The system then calculates the center coordinates of the detected box(es) for the previous image and the current image. The system then calculates the Euclidean distance between the center points of the box(es) and the center points of the current image’s box(es). Finally, the tracking ID of each box from the previous image is mapped on to the box(es) of the current image (Figure 10). In addition, the system adds new tracking items when new blooming flowers or immature fruits are detected. This process was repeated until the fruits grown on the truss were all approximately 3 cm or more in diameter (Figure 11). Then, monitoring the current truss was stopped, and a clip-type IoT camera was moved to the top truss. The tracking information of the blooming date and fruit numbers was then used to estimate the harvest dates and final yields.

#### 2.3.1. Temperature Forecasting

**Figure 12:**
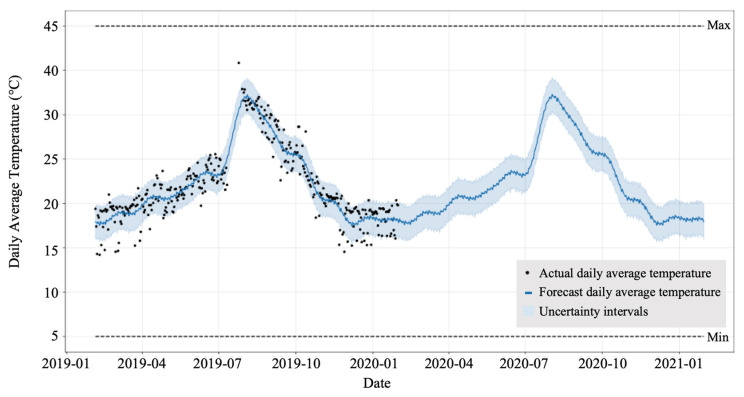
Daily average temperature prediction model.

In our system, we used temperature forecasting to estimate the harvest date of the blooming flower. For the forecasting, we used the Prophet package (https://facebook.github.io/prophet, accessed on 12 March 2022), which supports an additive model-based nonlinear regression with yearly, weekly, and daily seasonality including holiday effects. Moreover, the model is robust to outliers and missing data [28]. The temperature data acquired at our greenhouse also had outliers and missing data (due to power outages), thus we used the package for our dataset. Temperature sensors (Agrilog, ITKOBO-Z Co., Ltd., Nagoya, Japan) were installed in the greenhouse, and data were recorded every 5 min. The raw temperature data were processed to the average daily temperature. We generated a forecasting model using pre-processed daily average data. Our system predicted the average daily temperature after the date of flower blooming using the generated model (Figure 12).

#### 2.3.2. Harvest Prediction

Harvest prediction includes both harvest date estimation and the number of fruits to be harvested on the harvest date. The harvest date at each truss was calculated using the flower blooming date and integrated temperature. The integrated temperature was defined as the sum of the daily average temperature from the date of flower blooming (Equation (6))

(6) $$\mathrm{Integrated}\;\mathrm{Temperature}=\sum_{i=0}^{n}T_{d+i},$$

where *T* is the daily average temperature on date ‘*d* + *i*’. The ‘*d*’ indicates the date of flower blooming, while ‘*d* + *n*’ indicates the estimated harvesting date. In general, it is reported that tomato fruits can be harvested when the integrated temperature reaches 1100 °C after flower blooming [23,24,29]. It may differ among tomato cultivars; however, the ‘Momotaro York’ cultivar is known to have an integrated temperature of 1100 °C to 1200 °C [30,31].

Assuming that our system detects a blooming flower of the cultivar on ‘1 September 2020’, it assigns a tracking ID to the flower as ‘1_3_3’ (Figure 9). Then, the system predicts daily average temperature using a pre-trained temperature prediction model, and calculates the date when the ‘1_3_3’ flower reaches over 1100 °C. For example, we assume that every predicted daily average temperature from September to October is 20 °C. Then, the harvesting date is calculated as follows:

(7) $$1100{\;}^{{}^{\circ}}C\leq \overset{n}{\underset{i=0}{\sum }}T_{d+i}=20{\;}^{{}^{\circ}}C+20{\;}^{{}^{\circ}}C+20{\;}^{{}^{\circ}}C+\ldots +20{\;}^{{}^{\circ}}C\leq 1200{\;}^{{}^{\circ}}C$$

(8) $$1100{\;}^{{}^{\circ}}C\leq 20{\;}^{{}^{\circ}}C\left(n+1\right)\leq 1200{\;}^{{}^{\circ}}C$$

(9) $$\frac{1100{\;}^{{}^{\circ}}C}{20{\;}^{{}^{\circ}}C}-1\leq n\leq \frac{1200{\;}^{{}^{\circ}}C}{20{\;}^{{}^{\circ}}C}-1\to 54\;days\leq n\leq 59\;days$$

Then, the estimated possible harvesting date from the blooming date (i.e., 1 September 2020) was calculated as ‘25–30 October’ (Equations (7)–(9)).

In addition, our system calculates the number of fruits to be harvested by tracking from flower anthesis to specific fruit sizes. The system detects the number of tomato fruits that fall off the plant before ripening and flowers that have not become fruits. Based on these data, the system finally calculates the number of tomato fruits to be harvested on the estimated harvest date.

## 3. Results

### 3.1. Object Detection Using a Clip-Type IoT Camera

YOLO-based object detection was used for the flower and fruit detection networks. Light backbone networks such as Tiny and general backbone networks such as DarkNet19 or Darknet53 [32] were used in our experiments, which can be used for low-cost edge computer-based inferences.

**Figure 13:**
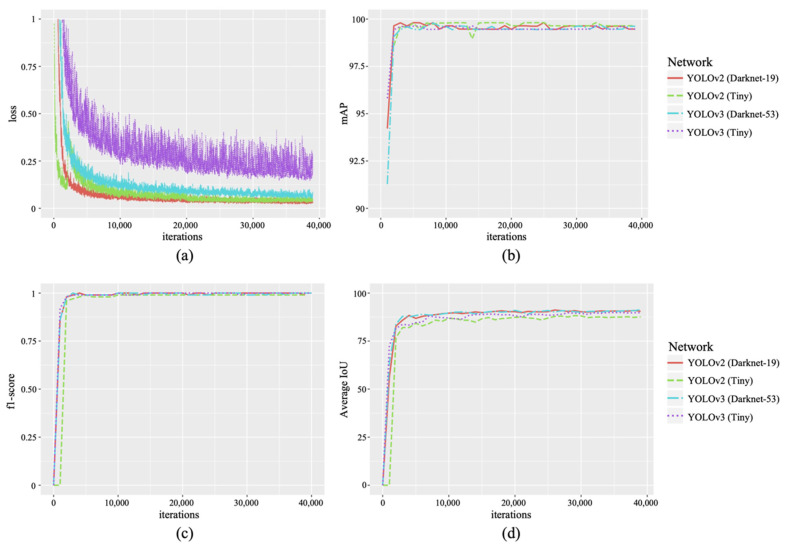
Validation results of bloomed flowers and immature fruit detection: (**a**) loss; (**b**) mAP; (**c**) f1-score; and (**d**) average IoU. mAP: Mean Average Precision; IoU: Intersection over Union.

In the experiments, 1350 images of flowers and fruits captured using the clip-type IoT camera in the greenhouse horticulture were used, and the ground truth for the two classes (i.e., fully opened flowers and immature fruits) was generated from the images. Ninety percent of the data were used as training data, and 10 percent of the data were used as validation data. The YOLO loss, F1-score, average IoU, and mAP (Figure 13) of the validation data were measured as metrics for each backbone network shown in Figure 8 and were measured using a desktop with Intel^®^ Core^TM^ i7-9700K CPU @ 3.60 GHz, 64 GB RAM, and NVIDIA GeForce RTX 2080 Ti 11 GB (HP Japan Inc., Tokyo, Japan).

The loss of each network decreased with the number of iterations. However, YOLO v3-Tiny showed slow and unstable training results. Each network showed a high mAP, F-1 score, and average IoU score after thousands of iterations. However, YOLO v2-Tiny showed a relatively low average score of IoU (Figure 13). In addition, the performance of the four YOLO models on our tomato validation dataset is presented in Table 1. The billion float operations per second (BFLOPS) describes the computing ability (low BFLOPS means faster operation) for the detection of each image using the graphics processing unit of the experimental desktop. The results showed that YOLO v2-Tiny and YOLO v3-Tiny provided low BFLOPS values, which are suitable for real-time tasks and low-cost edge computer devices.

### 3.2. Temperature Forecasting

The layout of the experimental greenhouse is shown in Figure 6. Four temperature sensors (i.e., T1 to T4) were installed at fixed positions. We trained four different temperature forecasting models using pre-processed daily average temperature data of each temperature sensor from February 2019 to January 2020. Each model was trained in the same hyperparameters [33] (i.e., interval_width = 0.95,growth = ‘logistic’, yearly_seasonality = True, changepoint_prior_scale = 0.01), but only the daily average temperature data used for training were different. The trained temperature models predicted the daily temperature until a set period. We predicted the daily average temperature from February 2020 to January 2021 using the trained models shown in Figure 12 and validated the predicted and actual data measured by the temperature sensors. To evaluate the performance of the trained models, we used the mean absolute error (MAE) metric shown in Equation (10), where *y_i_* is the predicted temperature, *x_i_* is the real temperature, and *n* is the number of dates:

(10) $$\mathrm{MAE}=\frac{\sum_{i=1}^{n}\left|y_{i}-x_{i}\right|}{n}$$

**Figure 14:**
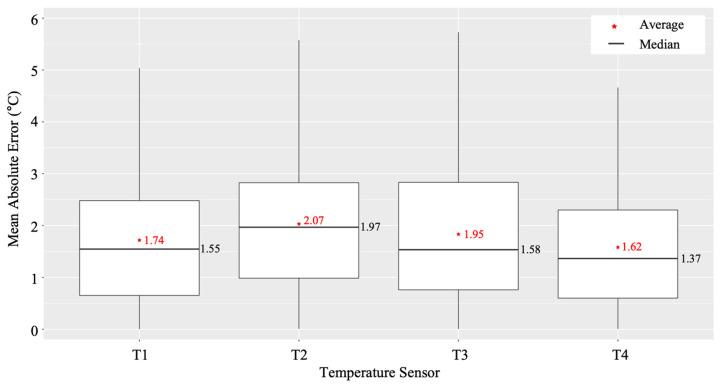
Mean Absolute Error (MAE) of each temperature sensor between actual daily average temperature and predicted daily average temperature.

**Figure 15:**
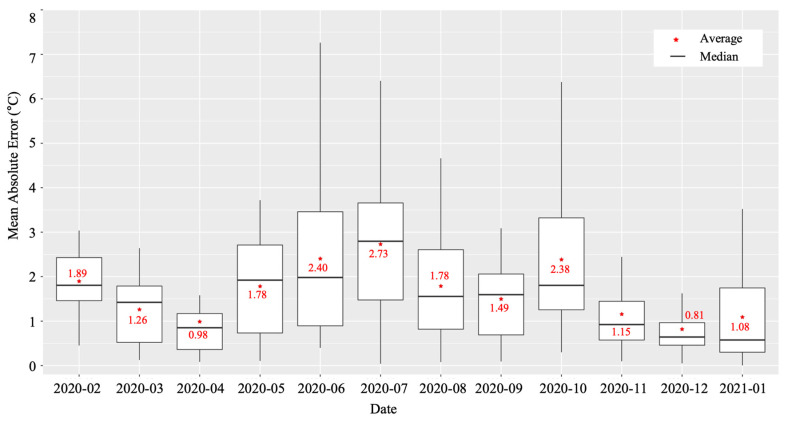
Monthly Mean Absolute Error (MAE) of the prediction model of the T4 temperature sensor.

We calculated the MAE of the four models from February 2020 to January 2021, as shown in Figure 14 and each model showed different performances according to the sensor positions in the greenhouse. The model of the T4 sensor showed the lowest error rate on average. In addition, the model of T4 showed a different MAE varying by month, as shown in Figure 15. The average MAE was lower from November to April than from May to October. Therefore, we adopted the forecasting model of the T4 sensor for the daily temperature prediction and integrated temperature calculation in the harvest prediction experiment.

### 3.3. Harvest Prediction

In this experiment, we evaluated the harvest date prediction ability of our system based on flower blooming detection, flowers and fruits tracking, and daily average temperature forecasting. For these experiments, we cultivated the Japanese tomato cultivar ‘Momotaro York’ from mid-August 2020 to the end of December 2020 at the Institute of Fruit Tree and Tea Science, NARO. The tomatoes were pruned to a single stem, and axillary buds were removed regularly.

**Figure 16:**
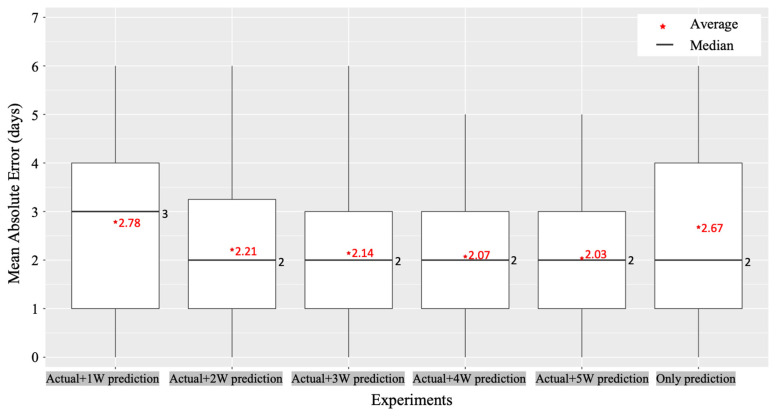
Average of Mean Absolute Error (MAE) between the actual harvest date and predicted harvest date.

**Figure 17:**
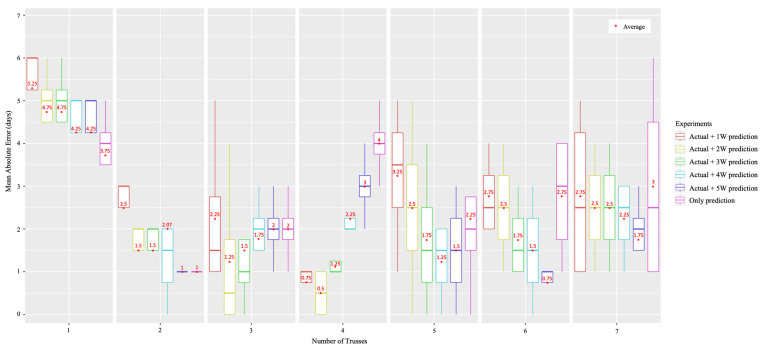
Mean Absolute Error (MAE) between the actual harvest date and predicted harvest date for each truss.

We configured a clip-type IoT camera system and sampled four different tomato plants in each line, as shown in Figure 6. The tomato samples were selected from the centerlines and not from the sidelines as they had a lot of disturbances from the external environment, and it was set as a sample with healthy growth and development in the early stage (Figure 3a). We monitored each tomato plant from the bud stage until the fruit size reached approximately 3 cm in diameter, and from the first truss to seventh truss using our system. In addition, we manually recorded the harvest date of each truss; then, the harvest date recorded manually and the harvest date predicted using our system were compared. We used the harvest date determined manually as a ground truth date (i.e., the correct answer). MAE was calculated between the actual and predicted harvest dates. We evaluated our system using six types of experiments, as shown in Figure 16. From the flower blooming date, we calculated the integrated daily average temperature using actual daily average temperature measured by a sensor and predicted daily average temperature a few weeks before (i.e., one to five weeks) the ground truth harvest date. As a result, the pair of actual daily average temperature and the predicted daily average temperature of five weeks before the ground truth harvest date showed the best performance. The difference between the ground truth harvest date and predicted harvest date was ±2.03 days. In addition, we analyzed the MAE for each truss. The first truss showed a high MAE. However, the second truss showed a low MAE (Figure 17).

The MAE was slightly different for each truss. However, on average, the harvest date could be predicted five weeks in advance with an error of approximately two days. This prediction can be used for greenhouse production management, such as labor management for tomato harvesting and harvest date control by temperature control.

## 4. Discussion

In our study, we used IoT cameras and self-designed clip-type accessories to monitor the growth and development of flowers and fruits in tomato plants. The clip-type IoT camera supported the accurate measurement of flowers and fruits. The harvest date was predicted via our system using the date of flower blooming and temperature forecasting. Experiments were conducted to verify the accuracy of the monitoring and prediction system, and the predicted harvest date determined using the flower blooming date was compared with the actual harvest date. It was found that our system could predict the harvest date with an average error of two days through this experiment. However, several factors can affect these errors. First, in the case of the temperature forecasting model used in this experiment, the lowest daily average temperature error was 1.62 °C (T4 sensor as shown in Figure 14). Thus, assuming that the daily average temperature inside the greenhouse is approximately 20 °C after blooming, it takes 55 days for fruit harvest after blooming (i.e., to reach the integrated temperature of 1100 °C). In addition, assuming that an error of ±1.62 °C in the daily temperature forecasting model occurs approximately every day for 55 days, the harvest date prediction error can be up to ±4.5 days. Secondly, there is an error caused by the actual harvest date (i.e., the ground truth). The actual harvest was not made on weekends or holidays, and the confirmation and harvesting were carried out only on Mondays, Wednesdays, and Fridays. For this reason, there is a possibility that an error of 1–2 days may have occurred. Finally, the fruit is usually harvested when the fruit is pink-colored, and there is a possibility that an error of 1–2 days may have occurred because of the subjective interpretation of the pink color. Therefore, sometimes, it was difficult to ensure that the actual harvest date is correct.

In the experiment, since power was supplied to the IoT camera via a wire, the movement of the clip-type IoT camera remains inconvenient depending on the growth or agricultural works of the crop. This may be solved by switching to a battery-powered IoT camera using a solar light rechargeable battery. It is possible to use IoT cameras with batteries that are compact and light because they require low power consumption. Thus, a battery can be mounted on the clip. Moreover, in some cases, occlusion of the camera had occurred because the growth rates of flowers and fruits were different and they moved over time, resulting in incorrect blooming detection. However, upon manual adjustment of the angle or position of the IoT camera, the occlusion disappeared. Finally, our system can be used in the field, but it has many limitations. This is because the field is greatly affected by the weather, so power supply problems and the accuracy of temperature prediction may decrease, and there is a limit to temperature control to adjust the harvest date.

## 5. Conclusions

This study proposes a system to monitor the growth and development of tomato truss and predict the harvest date. Our system monitors the blooming of tomato flowers as well as immature tomato fruits using deep learning-based object detection together with a self-designed clip-type IoT camera. In addition, the system predicts the harvest date using the blooming date of flowers and daily average temperature forecasting. Initially, we evaluated the monitoring and prediction performance of our system by conducting an experiment that compared the actual and predicted harvest dates.

We devised this clip since it was difficult to monitor the truss of tomato plants because the planting density was high in the greenhouse, the tomato plants grew rapidly, overlapping with other plants, and it was moved a lot due to agricultural works (e.g., movements of tomato guidelines). The clip was fixed to the stem and guide strings near the target truss of the tomato plant to monitor the truss from a short distance. As a result, we could acquire good images of the truss and easily monitor the plants. This minimizes errors of overlapping from obstacles (e.g., leaves) and errors in tracking the truss caused by movement (e.g., due to agricultural works).

The deep learning YOLO models were trained to detect blooming flowers and immature fruits using the data obtained by this device, and showed a high detection performance. This suggests that the date of flower blooming can be accurately recorded. Moreover, the results of experiments with simple and lightweight deep learning networks, such as YOLO-tiny, also showed high performance. Although the general YOLO model performs better than the YOLO-tiny model, there was no significant difference in their performance in our study. In other words, the dataset captured by our system made detection easier. Furthermore, simple and lightweight deep learning models such as YOLO-tiny can be mounted directly on IoT cameras (e.g., m5StickV) without additional deep learning hardware (i.e., a GPU).

We also evaluated the harvest prediction performance by comparing the predicted and actual harvest dates. It was difficult to determine the actual harvest date accurately in the experiments and the daily average temperature forecasting model had an error (i.e., 1.6 °C). However, on average, it was confirmed that the harvest date can be predicted with an error of ±2.03 days using our system. The yield schedule can be prepared by predicting the harvest date; thus, it can be used for production management, such as preparing the necessary manpower according to the yield schedule. In addition, it is possible to control harvest dates by controlling the temperature inside the greenhouse.

Furthermore, our system can be expected to make more accurate calculations and overall harvest date predictions if several clip-type IoT cameras are installed in a greenhouse because several tomato plants can be monitored simultaneously. Thus, prediction using our system can be expected to have applications in the precise management of growth and production of tomatoes.

## Figures and Tables

**Table 1:** Performance of each YOLO architecture.

YOLO Architectures	BFLOPS
**YOLO v3**	115.938
**YOLO v2**	52.083
**YOLO v3-Tiny**	9.673
**YOLO v2-Tiny**	12.329

YOLO: You only look once. BFLOPS: Billion float operations per second.
